# Genome-wide association analyses using a Bayesian approach for litter size and piglet mortality in Danish Landrace and Yorkshire pigs

**DOI:** 10.1186/s12864-016-2806-z

**Published:** 2016-06-18

**Authors:** Xiangyu Guo, Guosheng Su, Ole Fredslund Christensen, Luc Janss, Mogens Sandø Lund

**Affiliations:** Center for Quantitative Genetics and Genomics, Department of Molecular Biology and Genetics, Aarhus University, DK-8830 Tjele, Denmark

**Keywords:** GWAS, QTL region, Litter size, Piglet mortality, Pig

## Abstract

**Background:**

Litter size and piglet mortality are important traits in pig production. The study aimed to identify quantitative trait loci (QTL) for litter size and mortality traits, including total number of piglets born (TNB), litter size at day 5 (LS5) and mortality rate before day 5 (MORT) in Danish Landrace and Yorkshire pigs by genome-wide association studies (GWAS).

**Methods:**

The phenotypic records and genotypes were available in 5,977 Landrace pigs and 6,000 Yorkshire pigs born from 1998 to 2014. A linear mixed model (LM) with a single SNP regression and a Bayesian mixture model (BM) including effects of all SNPs simultaneously were used for GWAS to detect significant QTL association. The response variable used in the GWAS was corrected phenotypic value which was obtained by adjusting original observations for non-genetic effects. For BM, the QTL region was determined by using a novel post-Gibbs analysis based on the posterior mixture probability.

**Results:**

The detected association patterns from LM and BM models were generally similar. However, BM gave more distinct detection signals than LM. The clearer peaks from BM indicated that the BM model has an advantage in respect of identifying and distinguishing regions of putative QTL. Using BM and QTL region analysis, for the three traits and two breeds a total of 15 QTL regions were identified on SSC1, 2, 3, 6, 7, 9, 13 and 14. Among these QTL regions, 6 regions located on SSC2, 3, 6, 7 and 13 were associated with more than one trait.

**Conclusion:**

This study detected QTL regions associated with litter size and piglet mortality traits in Danish pigs using a novel approach of post-Gibbs analysis based on posterior mixture probability. All of the detected QTL regions overlapped with regions previously reported for reproduction traits. The regions commonly detected in different traits and breeds could be resources for multi-trait and across-bred selection. The proposed novel QTL region analysis method would be a good alternative to detect and define QTL regions.

**Electronic supplementary material:**

The online version of this article (doi:10.1186/s12864-016-2806-z) contains supplementary material, which is available to authorized users.

## Background

Reproduction, particularly female reproductive performance, is one of the most important components in livestock production. Litter size at weaning (LSW) has been considered as one of the most important reproduction traits in pig production [1]. In practical pig breeding, selection for total number of piglets born (TNB) was introduced in the early eighties in Danish Landrace and Yorkshire populations to improve LSW [2]. Unfortunately, this approach led to an increase in piglet mortality [1, 3–5]. In 2004 the breeding goal in the Danish breeding program was changed to focus on the litter size at five days after farrowing (LS5), and as result the mortality of piglets prenatally and in the early nursing period has decreased [6].

The genetic basis of reproductive performance is complicated because of the complex and quantitative nature of the traits. Using modern molecular information, many linkage [7–9] and candidate gene studies [10, 11] have been conducted to find the quantitative trait loci (QTL) and causal genes for these traits. More recently, the availability of high throughput genotyping makes it possible to study the genetic architectures and the genetic relationships of reproduction traits in pigs in further detail.

Based on the high-density panels of single nucleotide polymorphisms (SNP), genome-wide association studies (GWAS) have been developed to identify DNA variants associated with complex diseases and traits in humans and other animals [12]. GWAS has become a widely accepted approach to investigate genetic architectures of economically important traits in livestock. Many previous studies have carried out GWAS for complex traits in pig, such as teat number, androstenone and skatole levels, boar taint, backfat, loin muscle area, body conformation and brown coat color, and detected many QTLs for these traits [13–18]. Detection of QTL regions and genes affecting pig reproductive traits would be helpful for further understanding of these traits and genetic improvement of pig reproduction, but only few studies of reproductive traits have been made [19, 20].

Various approaches, such as single-marker tests [21], linear mixed model analysis [22], haplotype models and genealogy based mixed-models [23], Bayesian variable selection models [24], least absolute shrinkage and selection operator [25] have been proposed for GWAS. In previous model comparison studies, linear mixed model and Bayesian variable selection models were shown better than other methods in terms of detection power [22, 23, 26]. The linear mixed model, which is based on regression of phenotypes on SNP genotypes, is easy to implement. Each single marker is analyzed separately by using a linear model, which creates multiple testing problems as a large numbers of tests of SNP markers throughout the entire genome are performed. A multiple-testing problem which can lead to a high rate of type I errors could be created. Thus, a Bonferroni correction is often applied to set stringent thresholds on *P* values in order to avoid this problem, but this could result in poor statistical power. Besides, when many SNPs are in strong LD with one QTL, the use of a linear model makes it difficult to identify which SNP within a broad genomic region causally influences the complex trait and it is also troublesome to separate neighboring QTLs, which may contribute to the same peak. In addition, the linear model is also known for being sensitive for population and family structures, thus mixed models correcting for these effects are needed, either by adding pedigree or markers [26]. Therefore, it is appealing to apply Bayesian variable selection models, which simultaneously fit multiple marker effects, avoid multiple testing, and can implicitly correct for the structure [26, 27]. Additionally, the power to detect significant genetic association may be considerably enhanced by simultaneous modeling of markers. However, simultaneously fitting of SNP markers usually leads to low posterior probability for each SNP, in which case the sum of posterior probability of SNPs in a QTL region could be a better alternative option [28]. However, the sum of posterior probability of SNPs in a QTL region could be larger than 1 and it overestimates the posterior probability of a region.

Therefore, the objective of the current study was to identify QTL for the litter size and piglet mortality based on data from the PorcineSNP60 BeadChip in Danish Landrace and Yorkshire pigs. The performance of a Bayesian mixture model was compared with a linear mixed model and a novel method was proposed to detect QTL regions.

## Methods

### Data

The data from breeding herds and multiplier herds were supplied by Danish Pig Research Centre, SEGES P/S. Animal Care and Use Committee approval was not applicable for this study because the data were obtained from an existing database of pig breeding. Corrected phenotypes (*y*_*c*_) of TNB, LS5 and mortality rate before day 5 (MORT) were defined as original observations adjusted for effects of herd-year-season, parity, month at farrowing, hybrid indicator, age at first farrowing, parity-correction, farrowing interval, and artificial insemination. This methodology has previously been described by Guo et al. [29], in which litters of 545,124 Landrace and 361,978 Yorkshire pigs were used. In the present study, however, the populations used for computing *y*_*c*_ of both sows and boars were extended to younger generations by including additional litters of 235,762 Landrace and 171,218 Yorkshire sows.

Genotyping was done using the Illumina PorcineSNP60 BeadChip (Illumina, San Diego, CA) or imputed from the 8.5 K GGP-Porcine LD Illumina Bead SNP Chip. A total of 37,060 and 36,058 SNP markers for Landrace and Yorkshire pigs, respectively, met the following requirements. Each marker had a minor-allele frequency greater than 0.01, a call-frequency score greater than 0.9, average GenCall score larger than 0.60, no strong deviation from Hardy-Weinberg equilibrium (*P* > 10^−7^), and known position on Build 10.2 assembly (Sscrofa10.2). In addition, the animals with call rate less than 0.8 were excluded from the analysis. The imputation for lower density chips as well as sporadic missing genotypes were performed by using Beagle version 3.3.1 [30].

Finally, *y*_*c*_ of TNB, LS5 and MORT for 5,977 genotyped Landrace pigs (1,788 boars and 4,189 sows) and 6,000 genotyped Yorkshire pigs (1,761 boars and 4,239 sows) born from 1998 to 2014 were used for the analysis (around 10 percent of these animals were genotyped by the low density chip and imputed). Additional genotypes of 2,532 Landrace pigs (422 boars and 2,110 sows) and 2,628 Yorkshire pigs (520 boars and 2,108 sows) were added in this study, due to data updating.

### Statistical models

A linear mixed model and a Bayesian mixture model were used to perform GWAS in Danish Landrace and Yorkshire pigs separately to detect significant QTL associated with the three traits.

#### Linear mixed model (LM)

The LM model [22] used in this study was a single SNP regression model. The model included a fixed regression of phenotypes on genotypes of a given SNP, and in addition, a random polygenic effect accounting for shared genetic effects of related individuals. The LM model was:

$$ {\mathbf{y}}_{\mathbf{c}}=\mathbf{1}\mu +\mathbf{x}g+\mathbf{Z}\mathbf{u}+\mathbf{e}, $$

where **y**_**c**_ was the vector of *y*_*c*_ values of TNB, LS5 or MORT, *μ* was the overall mean, **1** was a vector of ones, *g* was the additive genetic effect of a SNP, **x** was a vector of the SNP genotypes coded as 0, 1, 2 for genotypes A_1_A_1_, A_1_A_2_ and A_2_A_2_ respectively, **u** was a vector of random polygenic effects, **Z** was an incidence matrix relating **y**_**c**_ to the corresponding random polygenic effects, and **e** was a vector of residual effects. It was assumed that $\mathbf{u}\sim \mathbf{N}\left(\mathbf{0},\mathbf{A}{\sigma}_u^2\right)$ where **A** was the pedigree-based additive relationship matrix and ${\sigma}_u^2$ was the variance of residual polygenic effect, and $\mathbf{e}\sim \mathbf{N}\left(\mathbf{0},\mathbf{D}{\sigma}_e^2\right)$, where ${\sigma}_e^2$ was the residual variance and **D** was a diagonal matrix containing the elements *d*_*ii*_ = 1/*w*_*i*_ where *w*_*i*_ was weight of *y*_*c*_ indicating the reliability of *y*_*c*_. The weight of *y*_*c*_ was calculated based on the reliability of *y*_*c*_ and as described in a previous study [29].

Significance test of SNP effects was performed using a two-sided *t*-test. A Bonferroni correction was applied to control false positive associations in a multiple comparison procedure. Thus, the significant level was defined as *P* < 0.05/N (or 0.01/N), where N was the number of SNP loci analyzed. Therefore, the significant threshold value of − log_10_(*P*) were 5.87 (6.57) and 5.86 (6.56) for Landrace and Yorkshire, respectively. Analysis of the LM model was performed by using the DMU package [31].

#### Bayesian mixture model (BM)

The BM model [24, 32, 33] used in the current study assumed SNP effects to follow a mixture distribution and estimated the effects of all SNPs simultaneously. The BM model was:

$$ {\mathbf{y}}_{\mathbf{c}}=\mathbf{1}\mu +{\displaystyle {\sum}_{j=1}^m{\mathbf{x}}_j}{g}_j+\mathbf{Z}\mathbf{u}+\mathbf{e}, $$

where **y**_**c**_, **1**, *μ*,** Z**, u and e were defined as in the LM model. The term ${\displaystyle {\sum}_{j=1}^m{\mathbf{x}}_j}{g}_j$ fitted additive effects of all SNPs, **x**_*j*_ was the vector of SNP_*j*_ genotypes, and *g*_*j*_ was the effect of SNP_*j*_. It was assumed that most markers had small effects and a few markers had large effects. Accordingly, the prior mixture distribution of *g*_*j*_ was:

$$ {g}_j\sim \left\{\begin{array}{c}\hfill N\left(0,{\sigma}_{g_0}^2\right)\hfill \\ {}\hfill N\left(0,{\sigma}_{g_1}^2\right)\hfill \end{array}\right.\begin{array}{c}\hfill\ \mathrm{with}\ \mathrm{probability}\ {\pi}_0\kern3em \hfill \\ {}\hfill\ \mathrm{with}\ \mathrm{probability}\ {\pi}_1=1-{\pi}_0,\hfill \end{array} $$

where *N* denoted normal distribution, *π*_0_ was the probability of the SNP having a small effect and *π*_1_ was the probability of the SNP having a large effect. It was assumed that the prior distribution of *π*_0_ and *π*_1_ was a Beta distribution with Beta (100, 1). Besides, it was assumed that priors of *μ* and ${\sigma}_{g_0}^2$ followed uniform distributions, and ${\sigma}_{g_1}^2={\sigma}_{g_0}^2\times 100$. By assuming a small variance instead of 0 for the distribution of $N\left(0,{\sigma}_{g_0}^2\right)$, the implementation of Markov Chain Monte Carlo (MCMC) was straightforward with recognizable conditional distributions for all model parameters [24, 32]. Each of the Bayesian analyses was run as a single chain with a total length of 52,000 Markov chain samples by Gibbs sampling, with the first 20,000 cycles discarded as burn-in. Afterwards, every 20th sample of the remaining 32,000 was saved for posterior analysis. Analysis of the BM model was performed by using the BayZ package [34].

### QTL region based on BM

The QTL region was detected by using a novel post-Gibbs analysis which was based on the MCMC samples. First, each chromosome was divided into many small sliding windows of equal length (1.0, 2.5 or 5.0 Mb) and a posterior probability of interval (*PP*_*int*_) was calculated according to the saved MCMC samples of all the markers in each window. In each window, *PP*_*int*_ was defined as the proportion of samples where at least one SNP within the window was falling into the second distribution (large effect) to total number of samples. Secondly, the peaks including windows with *PP*_*int*_ higher than the significant threshold (0.8) were chosen as candidate peaks to be further analyzed. In this study, the threshold of 0.8 was chosen. The threshold can be chosen by the investigator and directly reflect the posterior probability of a QTL in the region. Finally, the window with highest *PP*_*int*_ in each candidate peak was chosen as the QTL region. The genetic variance explained by a QTL region was computed as $\operatorname{var}\left({\mathrm{X}}_{\mathrm{region}}{\mathrm{b}}_{\mathrm{region}}^{\mathrm{t}}\right)$, which was the variance at the cycle t explained by the region and then the posterior mean and standard deviation were obtained.

## Results

### Detection of SNPs associated with reproductive traits

**Fig. 1:**
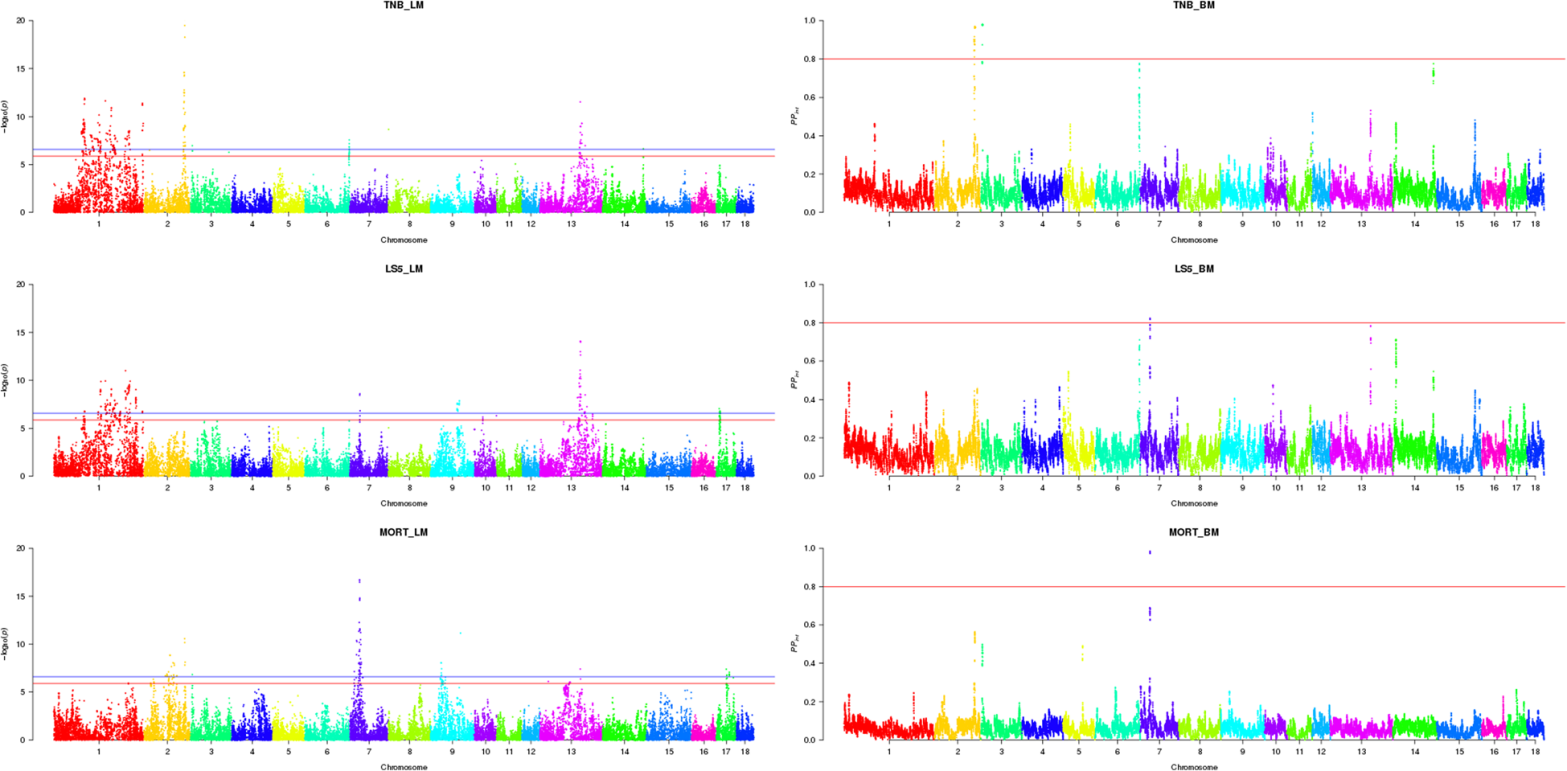
Manhattan plot of genome-wide association for reproduction traits in Landrace. Three plots on the left side are genome-wide *P*-values from a linear mixed model (LM) with single SNP regression for TNB, LS5 and MORT. The horizontal red and blue lines represent the genome-wide significance threshold at *P* < 0.05 and *P* < 0.01, respectively. Three plots on the right side are posterior probability of interval (*PP*_*int*_) for 1.0 Mb sliding window from a Bayesian mixture model (BM) QTL region analysis for TNB, LS5 and MORT. The horizontal red line represents the significance threshold at *PP*_*int*_ > 0.8

**Fig. 2:**
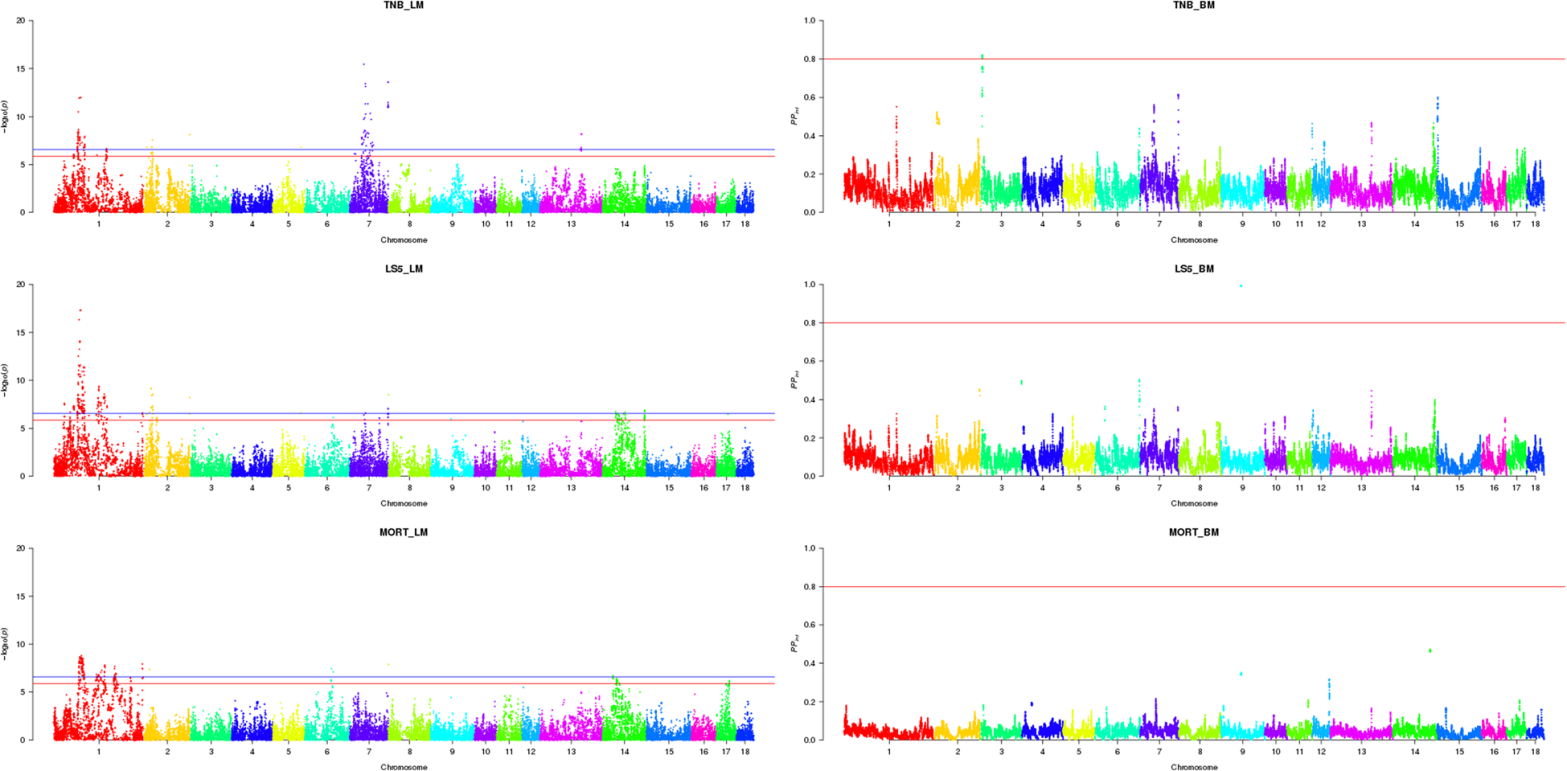
Manhattan plot of genome-wide association for reproduction traits in Yorkshire. Three plots on the left side are genome-wide *P*-values from a linear mixed model (LM) with single SNP regression for TNB, LS5 and MORT. The horizontal red and blue lines represent the genome-wide significance threshold at *P* < 0.05 and *P* < 0.01, respectively. Three plots on the right side are posterior probability of interval (*PP*_*int*_) for 1.0 Mb sliding window from a Bayesian mixture model (BM) QTL region analysis for TNB, LS5 and MORT. The horizontal red line represents the significance threshold at *PP*_*int*_ > 0.8

The LM model was used to perform single SNP test. The association patterns of SNPs with TNB, LS5 and MORT in Landrace and Yorkshire pigs using the LM model are shown in Figs. 1 and 2, respectively. The red line and the blue line represent the significant level after Bonferroni correction at *P* < 0.05 and *P* < 0.01, respectively. SNPs that had significant association with traits of interests can be visually observed in some chromosomes from the Manhattan plots. Table 1 shows the number of significant SNPs detected in each chromosome. Among all the chromosomes, the most significant SNPs can be found in chromosome 1 for all the traits in both breeds analyzed except MORT in Landrace where chromosome 7 embraced most significant SNPs.

**Table 1:** Number of significant SNPs detected using a linear mixed model with single SNP regression (LM)

Breed	Trait^a^	*P*	Total	Chromosome											
				1	2	3	5	6	7	8	9	10	13	14	17
Landrace	TNB	0.05	411	322	45	4		14		1			24	1	
		0.01	290	226	38	1		5		1			18	1	
	LS5	0.05	330	231					5		18	3	55		18
		0.01	206	140					3		18		37		8
	MORT	0.05	162	1	44	1			61		32		9		14
		0.01	94		23	1			51		7		1		11
Yorkshire	TNB	0.05	201	117	12		1		61				10		
		0.01	137	72	5		1		51				8		
	LS5	0.05	415	301	26		1	1	13	1	1			70	1
		0.01	275	248	13		1		4	1				8	
	MORT	0.05	338	278	1			6		1				46	6
		0.01	187	181	1			2		1				2	

^a^ *TNB* total number of piglets born, *LS5* litter size at day 5 after birth, *MORT* mortality rate before day 5 (including stillbirth)

For Landrace, at the significant level *P* < 0.05 there were 411, 330 and 162 SNPs detected as significant SNPs for TNB, LS5 and MORT, respectively, and at *P* < 0.01 there were 290, 206 and 94 SNPs for three traits correspondingly. For Yorkshire, the numbers of SNPs at significant level *P* < 0.05 were 201, 415 and 338 for TNB, LS5 and MORT, respectively, and at significant level *P* < 0.01 reduced to 137, 275 and 187 correspondingly. Among all the significant SNPs, as shown in Table 2, some have been commonly detected in different traits in both breeds.

**Table 2:** Number of significant SNPs common in different traits^a^ detected using LM^b^

Trait 1	Trait 2	*P* < 0.05	*P* < 0.01
Landrace TNB	Landrace LS5	110	107
Landrace TNB	Landrace MORT	7	6
Landrace TNB	Yorkshire TNB	17	13
Landrace TNB	Yorkshire LS5	77	50
Landrace TNB	Yorkshire MORT	128	52
Landrace LS5	Landrace MORT	6	6
Landrace LS5	Yorkshire TNB	3	4
Landrace LS5	Yorkshire LS5	5	10
Landrace LS5	Yorkshire MORT	23	16
Landrace MORT	Yorkshire TNB	1	0
Yorkshire TNB	Yorkshire LS5	114	57
Yorkshire TNB	Yorkshire MORT	23	8
Yorkshire LS5	Yorkshire MORT	178	133

^a^ *TNB* total number of piglets born, *LS5* litter size at day 5 after birth, *MORT* mortality rate before day 5 (including stillbirth)

^b^ *LM* a linear mixed model with single SNP regression

### Analysis of QTL regions

The BM model was used to test all SNPs simultaneously. Three sets of length, 1.0, 2.5 and 5.0 Mb of sliding windows were tested. A new method of detecting QTL region by using a Bayesian post-Gibbs analysis was proposed. The patterns of *PP*_*int*_ for 1.0 Mb sliding window are shown in the right side of Fig. 1 and Fig. 2. Table 3 shows the number of QTL regions detected for each chromosome in different scenarios and more details of these regions are presented on the supplementary material (Additional file 1: Figure S1, Additional file 2: Figure S2, Additional file 3: Figure S3, Additional file 4: Figure S4, Additional file 5: Figure S5 and Additional file 6: Figure S6). QTL regions were detected for all the traits in both breeds except MORT in Yorkshire for which no significant region was detected. The narrowest region among the three window sizes was chosen as the QTL region for each trait. Within these regions annotated genes were compared for their function. The positions and lengths of QTL regions, the genetic variance accounted by QTL regions as well as the related genes were listed in Table 4. For TNB in Landrace, when using 1.0 Mb as the length of sliding windows, three QTL regions were detected with two located on SSC2 and one on SSC3 (Additional file 1: Figure S1). The two regions on SSC2 explained 1.00 % and 0.75 % of additive genetic variance and the one on SSC3 explained 0.76 %. When the length of window was increased to 2.5 Mb, three more QTL regions located on SSC6, SSC13 and SSC14 were detected and they explained 0.98 %, 0.74 % and 0.59 % of additive genetic variance, respectively. The number of QTL regions did not increase further when the length of windows were expanded to 5.0 Mb. For LS5 in Landrace, the number of QTL regions increased from one (on SSC7) to three (on SSC6, 7, 13) when the length of window increased from 1.0 Mb to 2.5 Mb, and further increased to four (on SSC 6, 7, 13, 14) when using windows of length 5.0 Mb (Additional file 2: Figure S2). The proportions of additive genetic variance explained by the regions on SSC6 (2.5 Mb), SSC7 (1.0 Mb), SSC13 (2.5 Mb) and SSC14 (5.0 Mb) were 0.36 %, 0.38 %, 0.84 % and 0.44 %, respectively. For MORT in Landrace, only one region on SSC7 was detected when using 1.0 Mb and 2.5 Mb windows, and one more on SSC2 was detected when window amplified to 5.0 Mb (Additional file 3: Figure S3). The 1.0 Mb region on SSC7 explained 1.99 % of additive genetic variance and the 5.0 Mb region on SSC2 explained 0.77 %. For TNB in Yorkshire, one region on SSC3 was detected when using 1.0 Mb and 2.5 Mb windows (Additional file 4: Figure S4) and the 1.0 Mb region explained 0.46 % of the additive genetic variance, and one more on SSC1 was detected when windows were expanded to 5.0 Mb which explained 0.88 % of the additive genetic variance. However, only one QTL region on SSC9 was detected for LS5 in Yorkshire, no matter which window length was used (Additional file 5: Figure S5) and the 1.0 Mb region explained 0.51 % of the additive genetic variance.

**Table 3:** Chromosome (and number) of QTL regions detected using posterior probability in different length of windows

	Trait^a^	1.0 Mb	2.5 Mb	5.0 Mb
Landrace	TNB	2(2), 3(1)	2(1), 3(1), 6(1), 13(1), 14(1)	2(1), 3(1), 6(1), 13(1), 14(1)
	LS5	7(1)	6(1), 7(1), 13(1)	6(1), 7(1), 13(1), 14(1)
	MORT	7(1)	7(1)	2(1), 7(1)
Yorkshire	TNB	3(1)	3(1)	1(1), 3(1)
	LS5	9(1)	9(1)	9(1)

^a^ *TNB* total number of piglets born, *LS5* litter size at day 5 after birth, *MORT* mortality rate before day 5 (including stillbirth)

**Table 4:** The position and length of QTL regions and the genes within the regions

	Trait^a^	SSC^b^	Position 1	Position 2	Length	Proportion^c^	Gene
Landrace	TNB	2	139,963,748	140,963,748	1.0	1.00 %	PDLIM4, SLC22A4, IRF1, IL-5, IL13, IL4, GDF9, LEAP2
		2	141,775,934	142,775,934	1.0	0.75 %	VDAC1, PPP2CA, UBE2B, CDKN2AIPNL, SAR1B, SEC24A, CAMLG, DDX46, TXNDC15
		3	6,342,416	7,342,416	1.0	0.76 %	KPNA7, ARPC1A, ARPC1B, MIR9796, ATP5J2
		6	154,583,417	157,083,417	2.5	0.98 %	KLF17, ST3GAL3, ELOVL1, CDC20, CLDN19, GUCA2A, GUCA2B
		13	140,304,656	142,804,656	2.5	0.74 %	HES1
		14	141,048,490	143,548,490	2.5	0.59 %	MCMBP, FGFR2, TACC2
	LS5	6	154,583,417	157,083,417	2.5	0.36 %	KLF17, ST3GAL3, ELOVL1, CDC20, CLDN19, GUCA2A, GUCA2B
		7	34,743,041	35,743,041	1.0	0.38 %	HMGA1, LOC100156657, NUDT3, RPS10, C7H6orf106, SPDEF, SNRPC, TAF11, ANKS1A
		13	139,777,016	142,277,016	2.5	0.84 %	HES1
		14	8,423,533	13,423,533	5.0	0.44 %	STC1, NEFL, GNRH1, PPP2R2A, ADRA1A, EPHX2, GULO, CLU, CCDC25, PBK, PNOC, ZNF395
	MORT	2	138,703,617	143,703,617	5.0	0.77 %	CSF2, PDLIM4, SLC22A4, IRF1, IL-5, IL13, IL4, GDF9, LEAP2, VDAC1, PPP2CA, UBE2B, CDKN2AIPNL, SAR1B, SEC24A, CAMLG, DDX46, TXNDC15, H2AFY, CXCL14, IL9
		7	34,743,041	35,743,041	1.0	1.99 %	HMGA1, LOC100156657, NUDT3, RPS10, C7H6orf106, SPDEF, SNRPC, TAF11, ANKS1A
Yorkshire	TNB	1	181,896,016	186,896,016	5.0	0.88 %	RAB11A, MIR339-2, MAP2K1, SNAPC5, SMAD3, CLN6, SPESP1, RPLP1
		3	5,484,217	6,484,217	1.0	0.46 %	KPNA7, ARPC1A
	LS5	9	70,252,726	71,252,716	1.0	0.51 %	BTG2, OPTC, SNRPE, KISS1

^a^ *TNB* total number of piglets born, *LS5* litter size at day 5 after birth, *MORT* mortality rate before day 5 (including stillbirth)

^b^Pig chromosome

^c^The proportion of additive genetic variance explained by QTL region

Comparing the QTL regions for three traits in both breeds, there were some regions commonly detected for different traits. The common regions shared by different traits in both breeds are shown in Table 5. In Landrace, the two regions with 1.0 Mb located on SSC2 detected for TNB overlapped with the 5.0 Mb region detected for MORT. For TNB, on SSC3 the 1.0 Mb region detected for Landrace overlapped with the 1.0 Mb region detected for Yorkshire. In addition, for TNB and LS5 in Landrace two 2.5 Mb regions located on SSC6 and two 2.5 Mb regions located on SSC13 overlapped with each other, respectively. Finally, for LS5 and MORT in Landrace the two 1.0 Mb regions located on SSC7 overlapped.

**Table 5:** The QTL regions common in different traits^a^

SSC^b^	Breed 1	Trait 1	QTL region 1			Breed 2	Trait 2	QTL region 2		
			Position 1	Position 2	Length			Position 1	Position 2	Length
2	Landrace	TNB	139,963,748	140,963,748	1.0	-	MORT	138,703,617	143,703,617	5.0
2	Landrace	TNB	141,775,934	142,775,934	1.0	-	MORT	138,703,617	143,703,617	5.0
3	Landrace	TNB	6,342,416	7,342,416	1.0	Yorkshire	-	5,484,217	6,484,217	1.0
6	Landrace	TNB	154,583,417	157,083,417	2.5	-	LS5	154,583,417	157,083,417	2.5
7	Landrace	LS5	34,743,041	35,743,041	1.0	-	MORT	34,743,041	35,743,041	1.0
13	Landrace	TNB	140,304,656	142,804,656	2.5	-	LS5	139,777,016	142,277,016	2.5

^a^ *TNB* total number of piglets born, *LS5* litter size at day 5 after birth, *MORT* mortality rate before day 5 (including stillbirth)

^b^Pig chromosome

## Discussion

This study performed GWAS for litter size and mortality traits in Danish pigs. Two models, a linear model and a Bayesian mixture model, were used for the analysis. Many SNPs were detected to be significantly associated with the traits of interests. In addition, a novel approach to identify the QTL region was proposed. In total, 15 QTL regions were detected for TNB, LS5 and MORT in Danish Landrace and Yorkshire pigs.

### GWAS using LM and BM

The association patterns between markers and traits of interests were generally similar between the two models applied in the current study. Among 15 QTL regions identified by using BM, 12 regions embraced SNPs detected had significant association with traits of interests using LM. However the detection signals were more distinct when using BM than LM. The clearer peaks indicate that the BM model is better at identifying and distinguishing regions of putative QTL.

Population stratifications were investigated by calculating lambda-values [35]. The lambda-values found for Landrace were 1.50 (TNB), 1.76 (LS5), 1.71 (MORT), and 1.62 (TNB), 1.77 (LS5), 1.64 (MORT) for Yorkshire. The LM used in the current study implemented a polygenic component, based on the additive-relationship matrix, as an attempt to control the population stratifications. However, according to the lambda-values, the LM used could not control the population stratifications completely. The use of BM to fit all markers simultaneously in the model makes it possible to control the population stratifications [27].

Although the patterns of associations when using LM and BM models were generally consistent, some differences were observed. It was observed that many SNPs on SSC1 were detected significantly associated with most of the traits by using LM, while the BM model did not led to similar results, though there were some tentative peaks. The reason for the large amount of significant SNPs scattered across most of SSC1 when using LM is most likely assembly and map errors. The linkage disequilibrium (LD) pattern between significant SNPs was investigated. Strong LD was found between blocks of SNPs far away from each other and low LD was detected within block on SSC1. When compared with other chromosomes, SSC2 in both Landrace and Yorkshire showed several clear regions with strong LD between the SNPs within a region. However, the SNPs in LD with each other dispersed across the whole chromosome on SSC1. When SNPs in strong LD with a causal gene are wrongly mapped, LM may lead to peaks in the wrong location, while BM may not detect the QTL region because all markers are simultaneously fitted, which may make the *PP*_*int*_ of a region small if the SNPs in the region are wrongly mapped. When some assembly and map errors exist, LM and BM will lead to different results. In addition, the population stratifications could be another reason for the large amount of significant SNPs on SSC1 when LM was used. For SSC1, the lambda-values were 2.64 (TNB), 3.05 (LS5), 2.25 (MORT) for Landrace, and 2.41 (TNB), 3.97 (LS5), 3.59 (MORT) for Yorkshire. The lambda-values for SSC1 deviated more from 1 compared with the whole genome. However, when using BM, most of the significant SNPs detected by LM disappeared, which could also be due to good control of population stratifications by BM.

The differences in some significant chromosome segments between the two models can be explained by the different operation mechanism of models. When using LM, a single-locus regression analysis was performed, in which one SNP was fitted and a QTL effect was explained by a single SNP in each analysis. Therefore, a number of SNPs in LD with the QTL will generally present significant effects. In contrast, the BM model estimates the effects of all SNPs simultaneously. Therefore a QTL effect might be represented either by a single SNP or distributed over several SNPs that were in strong LD with the QTL. In other words, the effect of the single QTL could be represented by several markers jointly [21]. Since all the SNPs were fitted in the model simultaneously when using BM, a statistic across a small region is a good criterion to detect the QTL region instead of a single SNP.

Regardless of the different models, the amount of significant SNPs and regions detected in Yorkshire pigs was smaller than in Landrace. Also, heritabilities and genetic variances of the traits were higher for Landrace than for Yorkshire. Lastly, reliability of *y*_*c*_ for Landrace is higher than for Yorkshire, due to the larger data set available for Landrace. These results indicate that QTL detection power was higher for Landrace than for Yorkshire.

### QTL regions

Detection of QTL regions was based on the results from MCMC sampling. The *PP*_*int*_ represented the probability of large effect SNPs included in each window. The window with highest *PP*_*int*_ in each significant peak was chosen as a QTL region. Three sets of sliding windows with length of 1.0 Mb, 2.5 Mb and 5.0 Mb, were tested to detect QTL regions. The number of QTL regions was generally increased when increasing the window length, at the cost of achieving a broad interval. The optimal window size may differ between studies or even among different QTL in the same study depending on the extent of LD between markers and QTL, the effect size, as well as the power of detection. In this study, ten window sizes were tested as a preliminary investigation in order to choose an appropriate size of sliding window to report (results not shown). The ten window sizes varied from 0.5 Mb to 5.0 Mb, with the increment of 0.5 Mb. When using the 0.5 Mb, most of the sliding windows presented a low *PP*_*int*_ and the peaks were confounded with the background. However, when the window size varied from 1.0 Mb to 2.5 Mb and from 2.5 Mb to 5.0 Mb, the patterns of different scenarios could be generally represented by 1.0 Mb and 2.5 Mb, respectively. Lastly, the *PP*_*int*_ showed no significant changes when the window was enlarged to 5.0 Mb. As regard to the balance of detection power and positioning of QTL, sliding window with 1.0 Mb was good to locate QTL in a narrow region, 2.5 Mb was appropriate to locate QTL with higher detection power, while 5.0 Mb could be the upper limit to define a QTL region because the *PP*_*int*_ for a window larger than 5.0 Mb did not increase (Additional file 1: Figure S1, Additional file 2: Figure S2, Additional file 3: Figure S3, Additional file 4: Figure S4, Additional file 5: Figure S5 and Additional file 6: Figure S6). In general, there is no need to increase size of window when the *PP*_*int*_ no longer increase with the increasing of window size.

All the QTL regions detected for TNB, LS5 and MORT in the current study overlapped with previously reported QTL regions associated with reproduction in pigs which can be found in Table 6. The QTL regions reported in previous studies were mainly associated with corpus luteum number [36–38], teat number [39, 40], non-functional nipples [41, 42], age at puberty [7, 43], litter weight [44] and embryo weight [45]. All of these traits are relevant for litter size or piglet mortality. For example, the corpus luteum is essential for establishing and maintaining pregnancy in pigs [46]. Progesterone secreted by corpus luteum is a steroid hormone responsible for the decidualization of the endometrium and maintenance. Besides, genetic association between teat number and litter traits was investigated where a high number of non-functional teats was found genetically associated with more stillborn piglets [47].

**Table 6:** The QTL regions detected in current study overlapped with previously reported ones

	Current study		Previous studies		
SSC^a^	Position 1	Position 2	Position 1	Position 2	Trait
1	181,896,016	186,896,016	105,873,529	290,457,491	teat number [39]
			165,580,236	257,944,209	nonfunctional nipples [41]
			183,913,205	184,015,342	total number born [48]
			183,913,205	184,015,342	total number born alive [48]
			184,917,301	185,121,617	corpus luteum number [36]
			185,717,517	185,784,945	corpus luteum number [36]
2	138,703,617	143,703,617	116,028,974	145,137,405	corpus luteum number [36]
	139,963,748	140,963,748	138,002,690	142,235,710	nonfunctional nipples [41]
	141,775,934	142,775,934	139,070,668	139,947,117	age at puberty [43]
			139,359,663	150,135,089	nonfunctional nipples [42]
			139,525,787	139,963,748	mummified pigs [20]
			139,841,202	139,989,204	corpus luteum number [36]
			140,050,554	140,262,094	total number born [20]
			140,443,704	140,934,348	teat number [40]
3	5,484,217	6,484,217	1,456,046	130,209,174	teat number [39]
	6,342,416	7,342,416	4,571,903	82,495,610	corpus luteum number [37]
6	154,583,417	157,083,417	19,536,155	157,765,593	teat number [39]
7	34,743,041	35,743,041	8,014,191	92,220,281	corpus luteum number [38]
			10,763,543	117,929,721	teat number [39]
			11,136,187	116,028,974	corpus luteum number [36]
			16,365,408	42,509,154	age at puberty [7]
			34,692,239	35,120,284	total number born [44]
			34,692,239	35,120,284	total number born alive [44]
			34,692,239	35,120,284	litter weight, total [44]
9	70,252,726	71,252,726	39,568,811	138,751,051	corpus luteum number [37]
13	139,777,016	142,277,016	5,836,383	188,271,972	nonfunctional nipples [41]
	140,304,656	142,804,656	18,268,056	206,704,152	corpus luteum number [38]
			91,625,750	145,083,047	embryo weight [45]
			141,246,965	141,992,388	age at puberty [43]
14	8,423,533	13,423,533	12,814,009	12,929,926	total number born [20]
	141,048,490	143,548,490	141,246,965	141,992,388	age at puberty [43]

^a^Pig chromosome

In addition, some of the QTL regions reported in previous studies were specifically associated with litter size and mortality traits. For example, the QTL region located on SSC1 identified for TNB overlapped with the regions previously reported for TNB and total number born alive [48]. The QTL regions located on SSC2 identified for TNB and MORT overlapped with the regions reported for TNB and mummified pigs [20]. The QTL regions located on SSC7 identified for LS5 and MORT overlapped with the regions reported for TNB and total number born alive [44]. And the QTL regions located on SSC14 identified for TNB and LS5 overlapped with the region reported for TNB [20].

The overlap of QTL regions identified in this study with the regions reported in previous studies provides more confidence about the QTL regions detected.

### Candidate genes for reproduction traits

Some known genes were present on the QTL regions detected in this study, as it can be seen in Table 4. It was observed that most of the QTL regions cover a number of genes even though the region was narrow, e.g. the regions on SSC2 for TNB in Landrace. Therefore, pinpointing a specific candidate gene for such a QTL region is hard. Other regions cover one or two genes only, e.g. the region on SSC13 detected for TNB and LS5 in Landrace. This region only include the gene hairy and enhancer of split-1 (HES1, 140,633,462 ~ 140,635,357 bp). HES1 has been found to be involved in the maintenance of certain stem cells and progenitor cells, specifically influencing the timing of differentiation and determining binary cell fate. It has been shown that HES1 is playing a large role in both the nervous and digestive systems in mice [49, 50]. Another QTL region on SSC3 for TNB in Yorkshire included karyopherin alpha 7 (KPNA7, 6,330,984 ~ 6,364,374 bp). KPNA7, a member of the karyopherin α family of transport receptors, was first reported in cattle and was shown to be exclusively expressed in ovarian tissues, oocytes and cleavage stage embryos. RNAi-mediated knockdown of KPNA7 in bovine embryos lead to defects in cleavage development [51]. In addition, it was also reported that KPNA7 predominately expressed in porcine oocytes and early cleavage stage embryos, which suggests the requirement of KPNA7 for cleavage development [52]. Both HES1 and KPNA7 could be related with reproduction traits according to their function.

Though it is hard to pinpoint a specific candidate gene for QTL regions embracing several genes, these genes could influence a certain trait in a joint manner. Among the genes located on the detected QTL regions, many can be clustered in the group involved in process of cell growth, cell development, cell cycle arrest, cell differentiation and immune response etc. Some of the genes also have been reported in relation to specific reproduction processes. For example, the protein coded by sperm equatorial segment protein 1 (SPESP1, 184,819,956 ~ 184,820,069 bp) located on SSC1, is an acrosome membrane protein involved in sperm-egg binding and fusion [53]. The expression of SPESP1 has also been reported to be involved in the sperm–oocyte binding and fusion in pigs [54]. In addition, growth differentiation factor 9 (GDF9, 140,650,007 ~ 140,652,964) on SSC2 was the first oocyte-derived growth factor identified to be required for ovarian somatic cell function [55]. The GDF9 null mice were incapable of ovulation as a result of an arrest of follicle development at the primary stage, which indicated the essential role of GDF9 in folliculogenesis [55]. The expression of GDF9 was also investigated in pigs where expression of GDF9 was higher in oocytes than in cumulus/granulosa cells [56].

Among all the regions detected, the QTL region on SSC3 was commonly detected for TNB in both Landrace and Yorkshire. This region had positive effects on TNB in both breeds and the SNP located on 6,342,416 had relatively large effect which was found in the intron of the gene KPNA7. Therefore, this region and the KPNA7 gene could be good resource for across-breed selection. Another region located on SSC7 was commonly detected for LS5 and MORT in Landrace, while direction of the effects were opposite for these two traits. The effect of this QTL region was positive for LS5 but negative for MORT. The opposite effects of this region on LS5 and MORT could be part of the reason that negative genetic correlation between LS5 and MORT observed in Danish pigs [6]. As reported by previous studies, selection for TNB is generally associated with an increase of piglet mortality [1, 3, 4]. Accordingly, the Danish breeding program changed breeding goal from selection for TNB to LS5 in 2004, which has led to an increase in LSW and a decrease of piglet mortality [6]. The opposite effects of this region on these two traits also suggested the selection of LS5 is efficient to reduce MORT. There were some genes, e.g. high mobility group AT-hook 1 (HMGA1, 34,981,570 ~ 34,990,089 bp), nudix (nucleoside diphosphate linked moiety X)-type motif 3 (NUDT3, 35,003,934 ~ 35,018,877 bp), ribosomal protein S10 (RPS10, 35,109,882 ~ 35,117,473 bp), SAM pointed domain containing ets transcription factor (SPDEF, 35,214,393 ~ 35,233,199 bp) and small nuclear ribonucleoprotein polypeptide C (SNRPC, 35,451,476 ~ 35,466,721 bp), TAF11 RNA polymerase II, TATA box binding protein (TBP)-associated factor (TAF11, 35,543,524 ~ 35,555,701 bp) and ankyrin repeat and sterile alpha motif domain containing 1A (ANKS1A, 35,661,335 ~ 35,766,115 bp) involved in this region on SSC7 and some were reported associated with pig production traits such as backfat thickness, carcass length, foot weight, head weight [57] and growth traits such as limb bone length [58]. The common detection of this QTL region in several traits could provide good material to improve genomic models for multi-trait selection.

## Conclusions

This study revealed putative QTLs for TNB, LS5 and MORT in Danish Landrace and Yorkshire pigs. Compared with Bayesian models, the problem of population stratification cannot be considered sufficiently in the linear model. Bayesian models provided more precise peaks than linear mixed models. Using a novel approach, a total of 15 QTL regions were identified on SSC1, 2, 3, 6, 7, 9, 13 and 14 for three traits in both breeds. Among these QTL regions, 6 regions located on SSC2, 3, 6, 7 and 13 were associated with more than one trait. The QTL regions detected in the current study overlapped with the regions previously reported for reproduction traits.

## Additional files

Additional file 1: Figure S1. QTL region profiles for total number of piglet born (TNB) in Landrace in each chromosome. The horizontal red line represents the significance threshold at posterior probability of interval (*PP*_*int*_) > 0.8. The purple, blue and orange line represent the *PP*_*int*_ from a Bayesian model (BM) QTL region analysis based on 1.0 Mb, 2.5 Mb and 5.0 Mb sliding windows, respectively. (PNG 379 kb) Additional file 2: Figure S2. QTL region profiles for litter size at day 5 (LS5) in Landrace in each chromosome. The horizontal red line represents the significance threshold at posterior probability of interval (*PP*_*int*_) > 0.8. The purple, blue and orange line represent the *PP*_*int*_ from a Bayesian model (BM) QTL region analysis based on 1.0 Mb, 2.5 Mb and 5.0 Mb sliding windows, respectively (PNG 396 kb) Additional file 3: Figure S3. QTL region profiles for mortality rate before day 5 (MORT) in Landrace in each chromosome. The horizontal red line represents the significance threshold at posterior probability of interval (*PP*_*int*_) > 0.8. The purple, blue and orange line represent the *PP*_*int*_ from a Bayesian model (BM) QTL region analysis based on 1.0 Mb, 2.5 Mb and 5.0 Mb sliding windows, respectively. (PNG 292 kb) Additional file 4: Figure S4. QTL region profiles for total number of piglets born (TNB) in Yorkshire in each chromosome. The horizontal red line represents the significance threshold at posterior probability of interval (*PP*_*int*_) > 0.8. The purple, blue and orange line represent the *PP*_*int*_ from a Bayesian model (BM) QTL region analysis based on 1.0 Mb, 2.5 Mb and 5.0 Mb sliding windows, respectively. (PNG 390 kb) Additional file 5: Figure S5. QTL region profiles for litter size at day 5 (LS5) in Yorkshire in each chromosome. The horizontal red line represents the significance threshold at posterior probability of interval (*PP*_*int*_) > 0.8. The purple, blue and orange line represent the *PP*_*int*_ from a Bayesian model (BM) QTL region analysis based on 1.0 Mb, 2.5 Mb and 5.0 Mb sliding windows, respectively. (PNG 346 kb) Additional file 6: Figure S6. QTL region profiles for mortality rate before day 5 (MORT) in Yorkshire in each chromosome. The horizontal red line represents the significance threshold at posterior probability of interval (*PP*_*int*_) > 0.8. The purple, blue and orange line represent the *PP*_*int*_ from a Bayesian model (BM) QTL region analysis based on 1.0 Mb, 2.5 Mb and 5.0 Mb sliding windows, respectively. (PNG 249 kb)
