# Supplementary figures and images for: Genome-wide association analyses using a Bayesian approach for litter size and piglet mortality in Danish Landrace and Yorkshire pigs
Source: BMC Genomics. 2016 Jun 18;17:468. doi: 10.1186/s12864-016-2806-z (PMC4912826; doi:10.1186/s12864-016-2806-z)

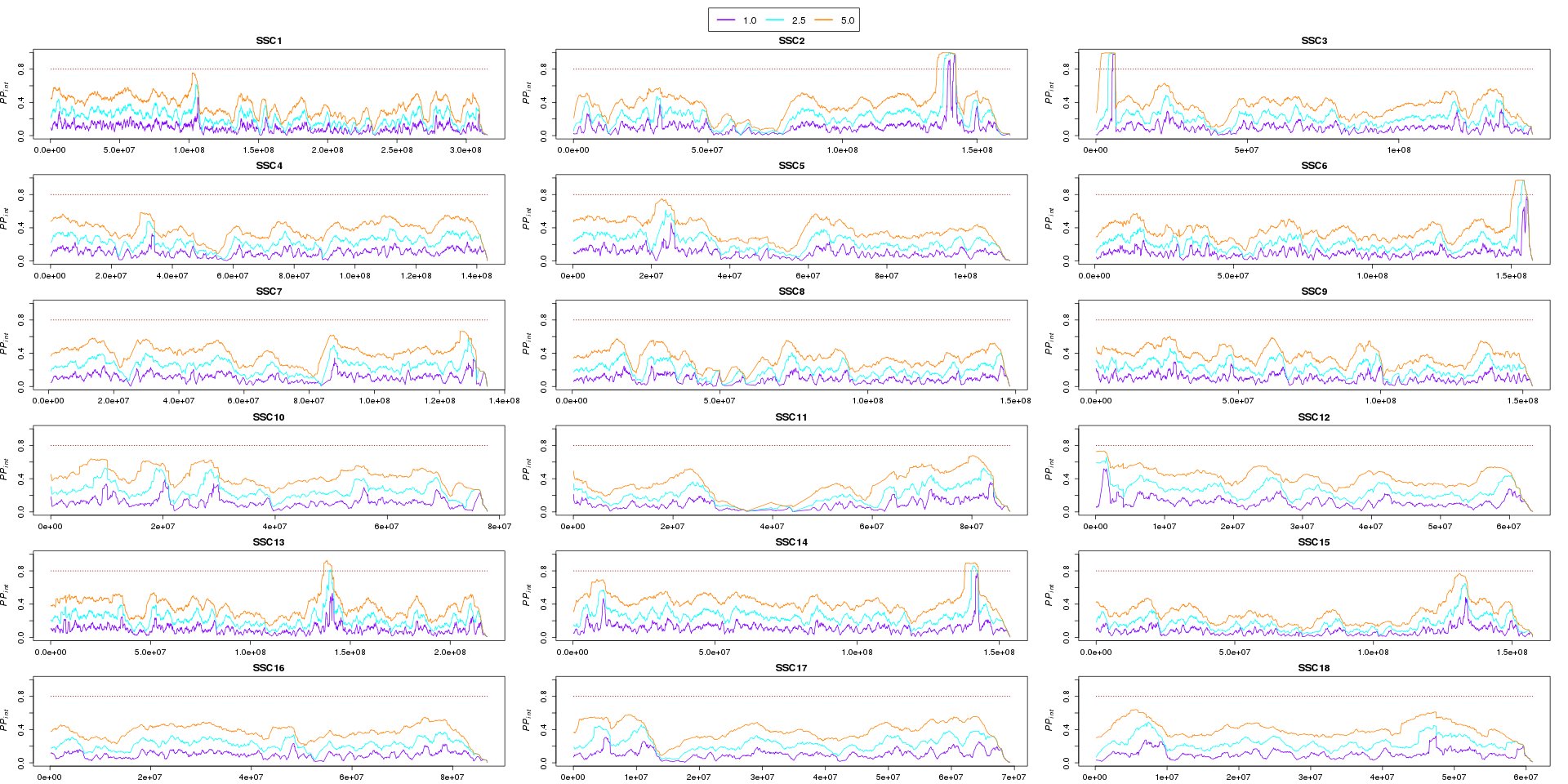

Supplement: Additional file 1: Figure S1. — QTL region profiles for total number of piglet born (TNB) in Landrace in each chromosome. The horizontal red line represents the significance threshold at posterior probability of interval (PP int) > 0.8. The purple, blue and orange line represent the PP int from a Bayesian model (BM) QTL region analysis based on 1.0 Mb, 2.5 Mb and 5.0 Mb sliding windows, respectively. (PNG 379 kb) [file 12864_2016_2806_MOESM1_ESM.png]

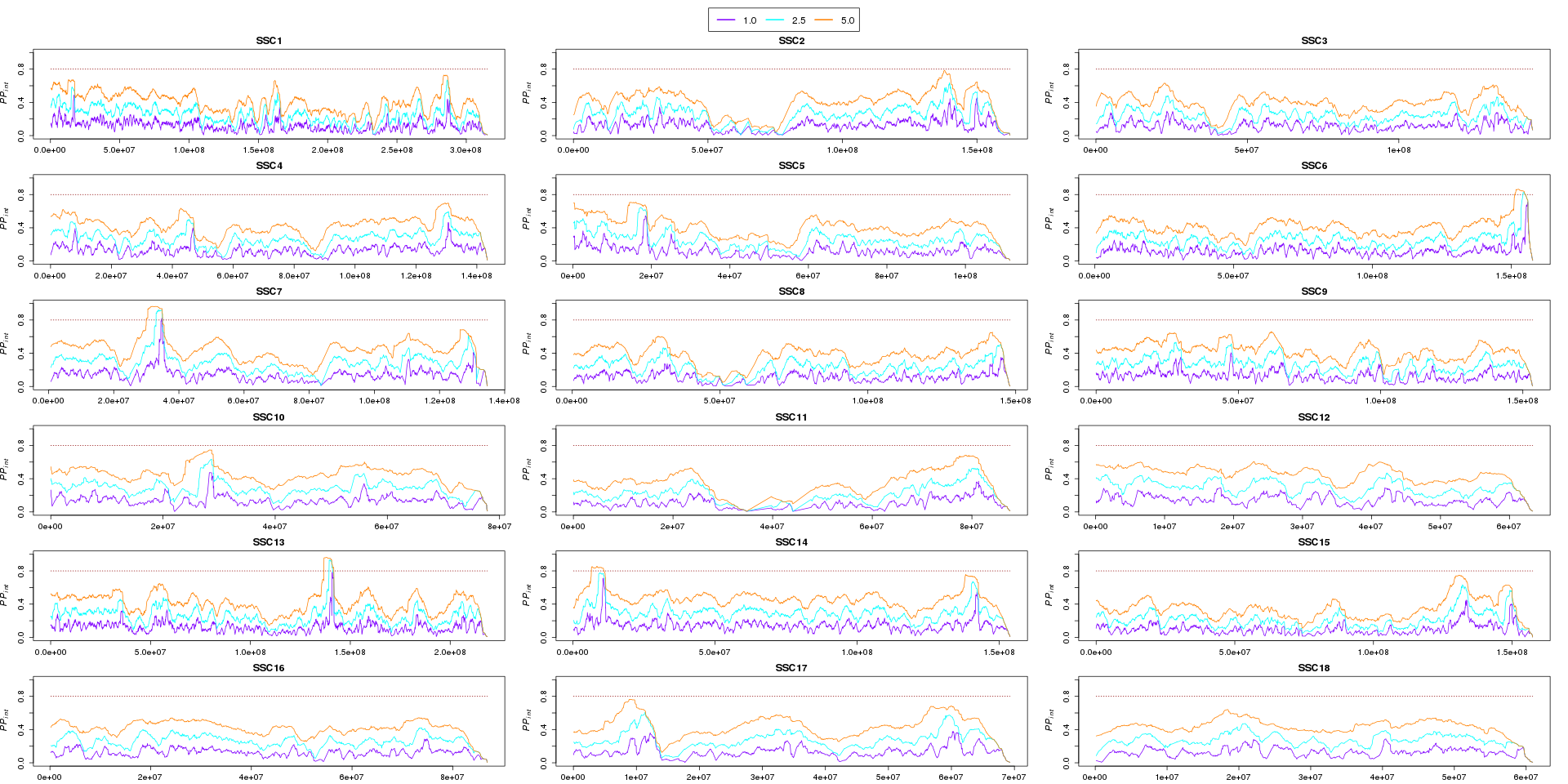

Supplement: Additional file 2: Figure S2. — QTL region profiles for litter size at day 5 (LS5) in Landrace in each chromosome. The horizontal red line represents the significance threshold at posterior probability of interval (PP int) > 0.8. The purple, blue and orange line represent the PP int from a Bayesian model (BM) QTL region analysis based on 1.0 Mb, 2.5 Mb and 5.0 Mb sliding windows, respectively (PNG 396 kb) [file 12864_2016_2806_MOESM2_ESM.png]

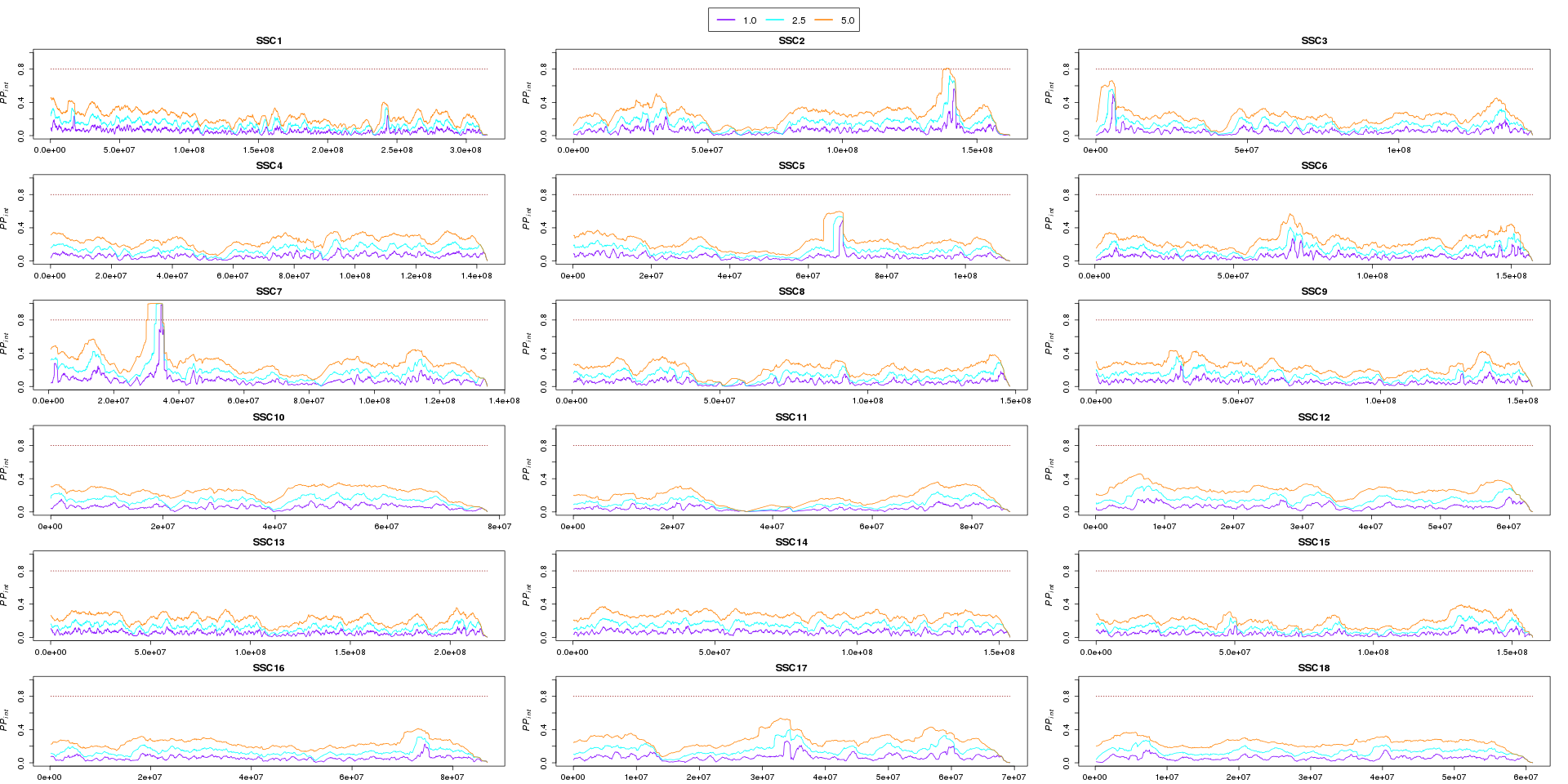

Supplement: Additional file 3: Figure S3. — QTL region profiles for mortality rate before day 5 (MORT) in Landrace in each chromosome. The horizontal red line represents the significance threshold at posterior probability of interval (PP int) > 0.8. The purple, blue and orange line represent the PP int from a Bayesian model (BM) QTL region analysis based on 1.0 Mb, 2.5 Mb and 5.0 Mb sliding windows, respectively. (PNG 292 kb) [file 12864_2016_2806_MOESM3_ESM.png]

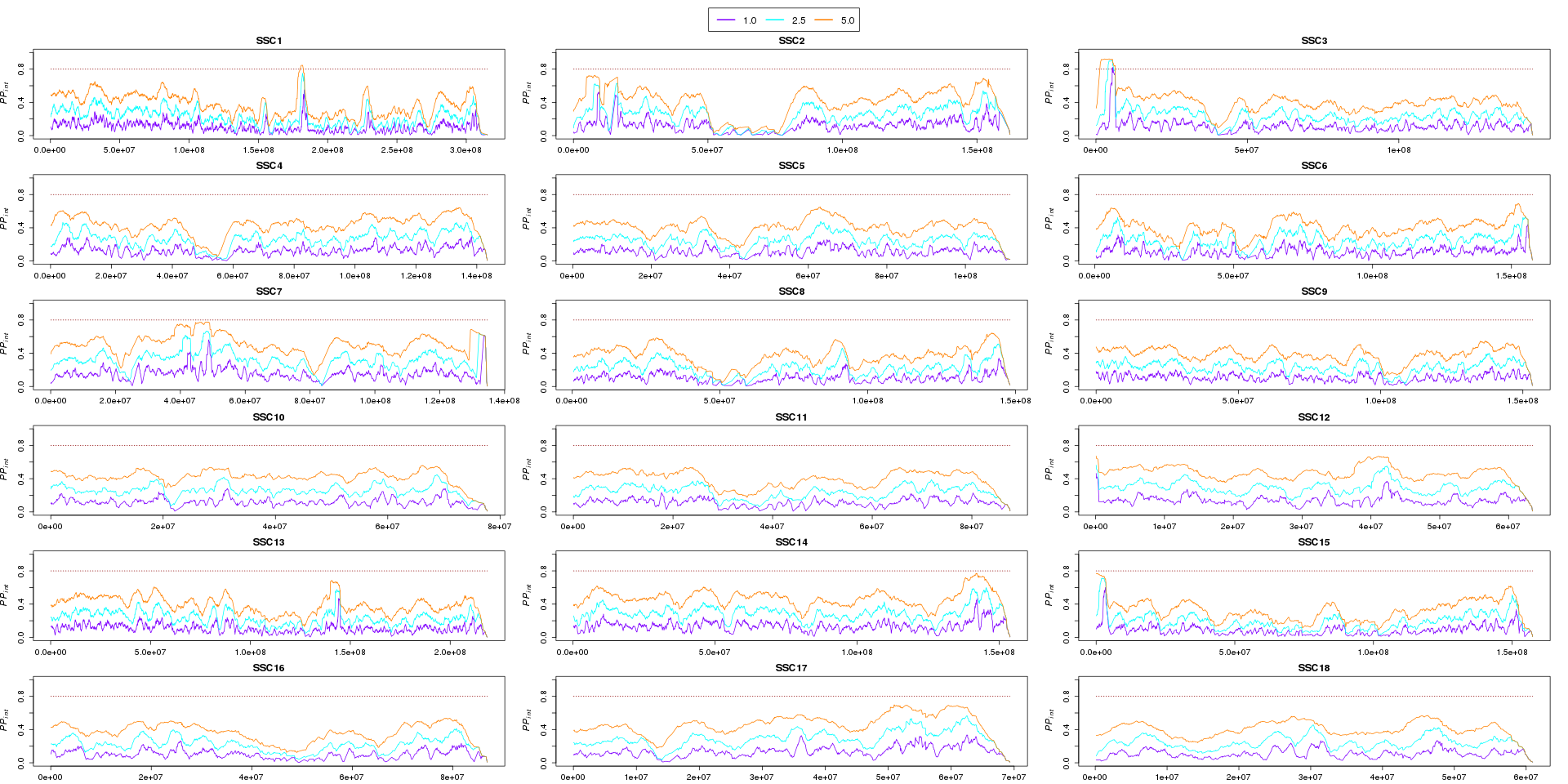

Supplement: Additional file 4: Figure S4. — QTL region profiles for total number of piglets born (TNB) in Yorkshire in each chromosome. The horizontal red line represents the significance threshold at posterior probability of interval (PP int) > 0.8. The purple, blue and orange line represent the PP int from a Bayesian model (BM) QTL region analysis based on 1.0 Mb, 2.5 Mb and 5.0 Mb sliding windows, respectively. (PNG 390 kb) [file 12864_2016_2806_MOESM4_ESM.png]

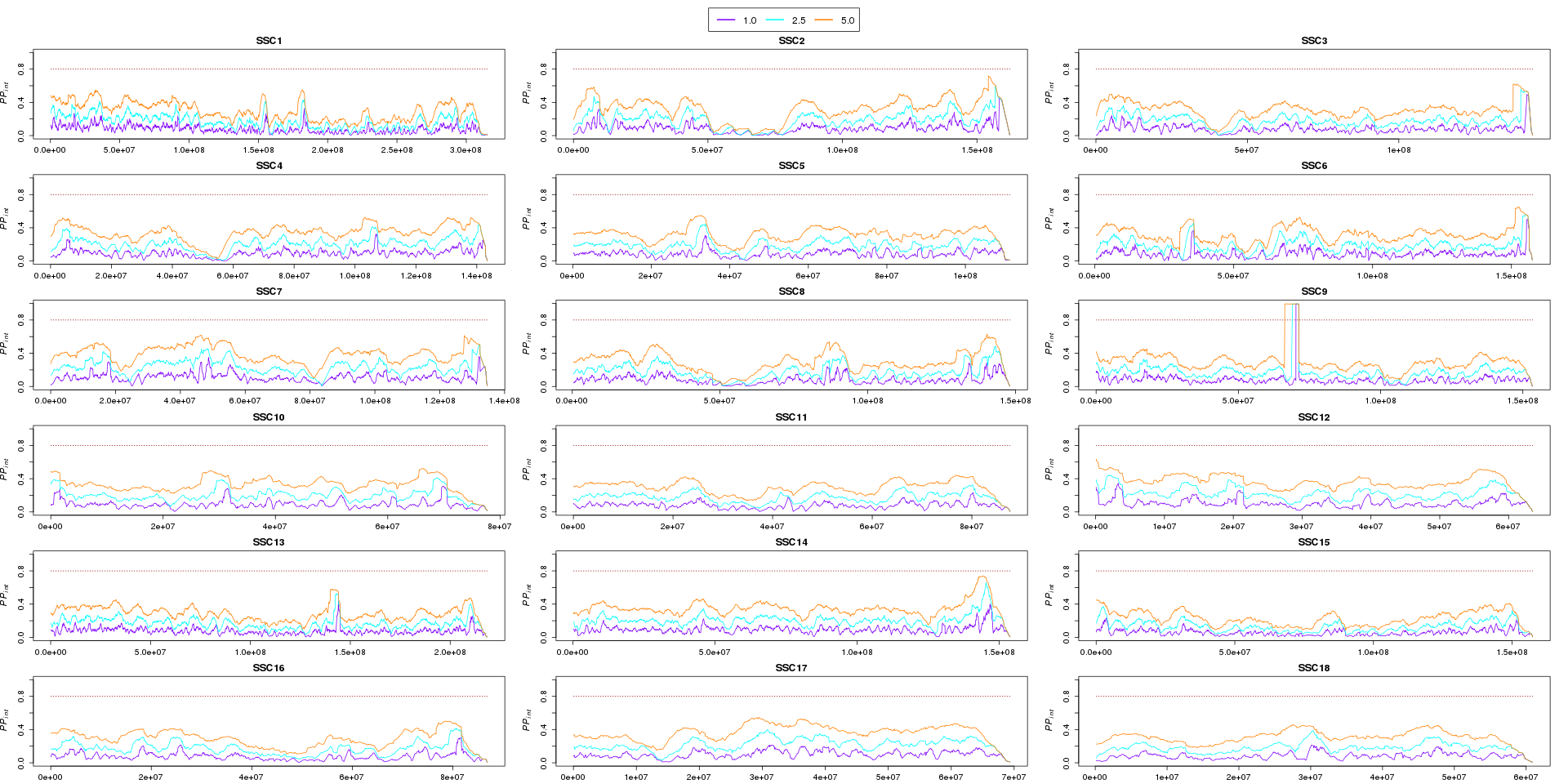

Supplement: Additional file 5: Figure S5. — QTL region profiles for litter size at day 5 (LS5) in Yorkshire in each chromosome. The horizontal red line represents the significance threshold at posterior probability of interval (PP int) > 0.8. The purple, blue and orange line represent the PP int from a Bayesian model (BM) QTL region analysis based on 1.0 Mb, 2.5 Mb and 5.0 Mb sliding windows, respectively. (PNG 346 kb) [file 12864_2016_2806_MOESM5_ESM.png]

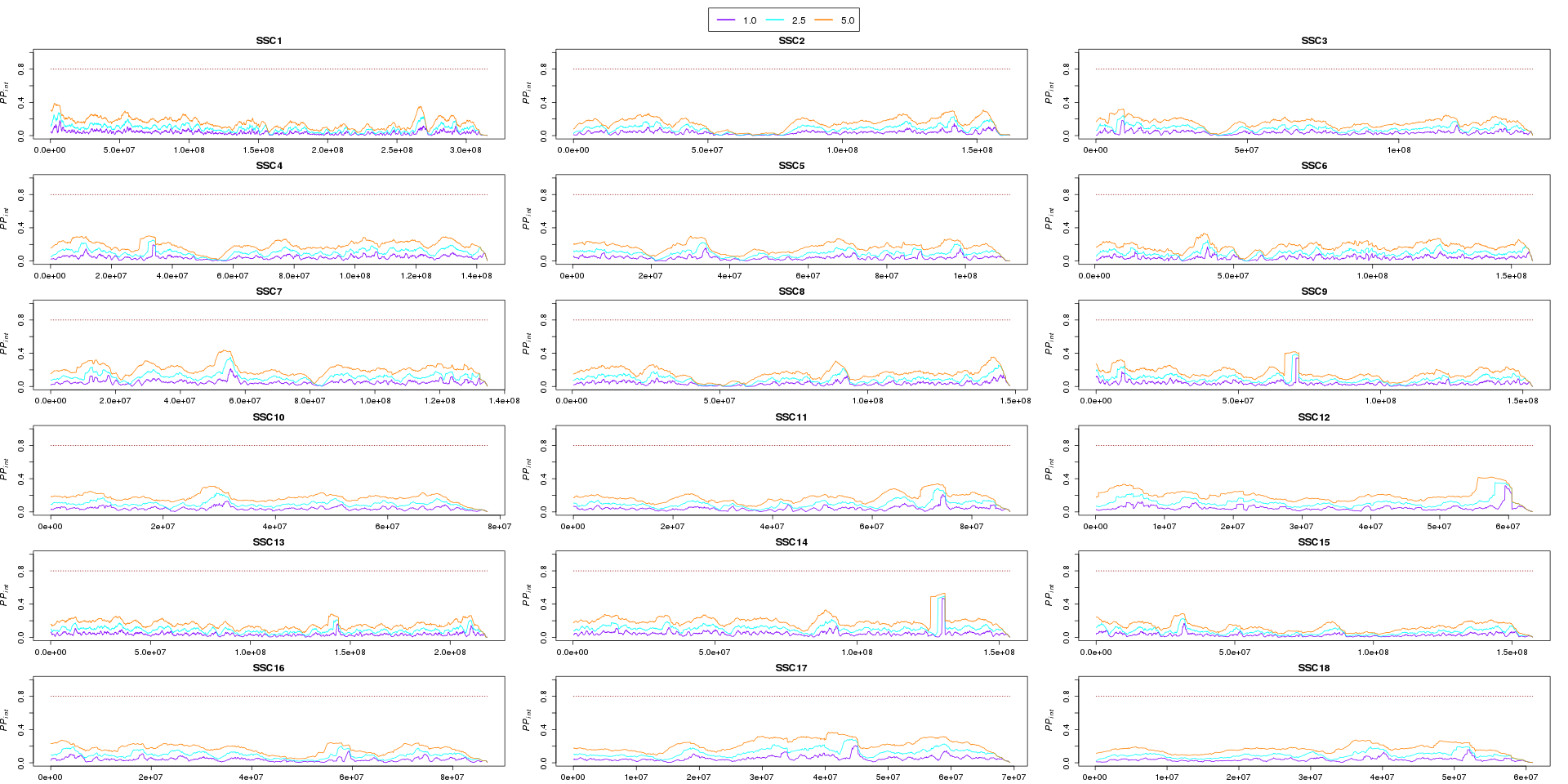

Supplement: Additional file 6: Figure S6. — QTL region profiles for mortality rate before day 5 (MORT) in Yorkshire in each chromosome. The horizontal red line represents the significance threshold at posterior probability of interval (PP int) > 0.8. The purple, blue and orange line represent the PP int from a Bayesian model (BM) QTL region analysis based on 1.0 Mb, 2.5 Mb and 5.0 Mb sliding windows, respectively. (PNG 249 kb) [file 12864_2016_2806_MOESM6_ESM.png]
